# Large-scale, prospective, observational studies in patients with psoriasis and psoriatic arthritis: A systematic and critical review

**DOI:** 10.1186/1471-2288-11-32

**Published:** 2011-03-31

**Authors:** Sue Langham, Julia Langham, Hans-Peter Goertz, Mark Ratcliffe

**Affiliations:** 1 PHMR consulting, London, UK; 2 Department of Public Health and Policy, Health Services Research Unit, London School of Hygiene and Tropical Medicine, London, UK; 3 Novartis Pharma AG, Health Economics & Outcomes Research, Basel, Switzerland

## Abstract

**Background:**

Observational studies, if conducted appropriately, play an important role in the decision-making process providing invaluable information on effectiveness, patient-reported outcomes and costs in a real-world environment. We conducted a systematic review of large-scale, prospective, cohort studies with the aim of (a) summarising design characteristics, the interventions or aspects of the disease studied and the outcomes measured and (b) investigating methodological quality.

**Methods:**

We included prospective, cohort studies which included at least 100 adults with psoriasis or psoriatic arthritis. Studies were identified through searches in electronic databases (Pubmed, Medline, Cochrane library, Centre for Reviews and Dissemination). Information on study characteristics were extracted and tabulated and quality assessment, using a checklist of 18 questions, was conducted.

**Results:**

Thirty five papers covering 16 cohorts met the inclusion criteria. There were ten treatment-related studies, only two of which provided a comparison between treatments, and six non-treatment studies which examined a number of characteristics of the disease including mortality, morbidity, cost of illness and health-related quality of life. All studies included a clinical outcome measure and 11 included patient-reported outcomes, however only two studies reported information on patient utilities and two on costs. The quality of the assessed studies varied widely. Studies did well on a number of quality assessment questions including having clear objectives, documenting selection criteria, providing a representative sample, defining interventions/characteristics under study, defining and using appropriate outcomes, describing results clearly and using appropriate statistical tests. The quality assessment criteria least adhered to involved questions regarding sample size calculations, describing potential selection bias, defining and adjusting for confounders and losses to follow-up, and defining and describing a comparison group.

**Conclusion:**

The review highlights the need for well designed prospective observational studies on the effectiveness, patient-reported outcomes and economic impact of treatment regimes for patients with psoriasis and psoriatic arthritis in a real-world environment.

## Background

Psoriasis is a chronic, non-contagious skin disease that commonly leads to appearance of red scaly patches on the skin. Psoriatic arthritis is a chronic, disabling inflammatory disease, associated with psoriasis. In psoriatic arthritis patients, the immune system attacks its own joints thus leading to joint destruction associated with cartilage deterioration, bone damage and joint fusion. Prevalence of the disease is around 2-3% of the world population. It causes considerable morbidity, significantly affecting the quality of life of those suffering from the disease[1-5]. Psoriasis is linked with psychological distress[6], depression[7,8], pain and physical disability[9]. In addition it carries significant economic implications, due to direct costs of management and costs associated with productivity losses[10-13]. Furthermore, there is some evidence to suggest that psoriasis and psoriatic arthritis may be associated with the development of heart disease, cancer and infections leading to premature death[14-17].

There are a number of systemic treatments for psoriasis and psoriatic arthritis which have been examined in numerous randomised controlled trials (RCTs)[18,19]. However, while RCTs are considered the gold standard for evidence-based decision making, it has been argued that observational studies have an important role in the measurement of effectiveness, longer-term outcomes, rare adverse events, and other outcomes requiring a more naturalistic study environment, for example the measurement of resource use and health-related quality of life (HRQOL)[20,21]. RCTs are generally designed to test efficacy and safety. Although efficacy and effectiveness both address the issue of whether a particular intervention works or not, efficacy assesses whether an intervention works under optimal circumstances, whereas effectiveness assesses whether an intervention works in usual care. Effectiveness is meant to be a more pragmatic measure that addresses the utility of a drug as it is actually employed in practice, therefore to measure effectiveness it is necessary to mirror a real-world environment as much as possible. RCTs often use narrow inclusion criteria and exclude patients with specific co-morbidities. In addition, sample sizes can be restricted and follow-up periods short. Such design characteristics mean that RCTs often have low external validity (how results can be generalised to the wider population) which limits their use in guiding treatment in routine clinical practice.

An observational study, by definition, is a study in which the investigators do not seek to intervene, only observe the course of events. Changes or differences in one characteristic (e.g. whether or not people received systemic treatment) are studied in relation to changes or differences in other characteristics (e.g. whether or not HRQOL improved), without action by the investigator. Such studies have high external validity but lower internal validity than RCTs. Results are more generalisable, but it is more difficult to attribute differences in outcomes between comparison groups to the particular intervention or characteristic under observation because of potential differences in baseline patient characteristics or because of losses to follow-up. It is important therefore for observational studies to be well designed and constructed and employ techniques to minimise the susceptibility of bias. Of the three types of observational study (cohort, cross-sectional and case-control), the cohort study stands at the top of the hierarchy of clinical observational evidence as it measures events in temporal sequence and can thereby more easily distinguish cause from effect. It is the most appropriate method to measure incidence of specific events, the natural history of the disease, changes in health states and use of healthcare resources.

Observational studies can play an important role in the decision-making process. The National Institute of Clinical Excellence stresses that decision-makers need to assess and appraise all the available evidence regardless of whether it has been derived from a RCT or an observational study. In the United States comparative effectiveness research (i.e. the direct comparison of existing health care interventions to determine which work best for which patients and which pose the greatest benefits and harms) assesses effectiveness in patients typical of day to day clinical care and therefore the focus is on 'real life' studies rather than RCTs. Such comparative effectiveness research is being employed by the government to improve the quality of health care whilst reducing the rising costs. Both approaches have their strengths and weaknesses and it is important for decision-makers to understand these when using the evidence to inform them of the appropriate use of interventions in routine clinical practice[22]. Response to treatment in patients with psoriasis is unpredictable and often patients become resistant. This leads to individualised treatment regimes. The restrictive nature of RCTs would not necessarily highlight the outcomes that would be seen usual clinical practice where patients are often exposed to a number of different treatment regimes before response is achieved. Also as some of the treatments are associated with potentially serious side-effects, longer-term observational studies can provide important additional information to a variety of stakeholders including clinicians, payers, providers and patients when weighing up the risks and benefits of treatment.

We carried out a comprehensive review of large-scale, prospective, cohort studies conducted on patients with psoriasis and psoriatic arthritis. Our primary aim was to (a) summarise the design characteristics, the interventions or aspects of the disease studied and the outcomes measured and (b) investigate the methodological quality of included studies.

## Methods

We included prospective, cohort studies which included at least 100 adults with psoriasis or psoriatic arthritis. We included 'treatment' studies that focused on a particular intervention, drug or group of drugs with any comparison and 'non-treatment' studies that assessed the impact on psoriasis or psoriatic arthritis on morbidity, mortality, resource use or HRQOL. We excluded all studies with an experimental element to them (RCTs, open-label studies and open-label extensions). We also excluded retrospective studies, cross-sectional studies, studies where patient's age was less than 18 years old and unpublished studies. We employed a cut-off of 100 patients to define large scale because (a) a recent health technology assessment of the management of psoriasis employed this cut-off for observational studies [23] and (b) other studies have used this as a cut-off to define large scale studies [24].

A systematic electronic literature search was conducted to identify published reports using the following databases; PUBMED (1965 to 2009), MEDLINE (1989 to 2009), Cochrane Library (which includes Cochrane reviews, other reviews, clinical trials, methods studies, technology assessments and economic evaluations), the Centre for Reviews and Dissemination Database (which includes the Database of Abstracts of Reviews of Effects, NHS Economic Evaluation Database, Health Technology Assessment Database) and one internet search engine (google). Additionally the following databases were reviewed to search for ongoing and planned studies; TRIP Turning Research into Practice database, National research register, ClinicalTrials.gov, Current Controlled Trials, Early Warning System and Salford database of psoriasis trials. Search terms combined disease terms (psoriasis and psoriatic arthritis) with study types (cohort, epidemiologic, follow-up, longitudinal, prospective, registries, Phase IV, observational). Studies were restricted to those in humans and in the English language. For example the search string used in PUBMED and MEDLINE was ("Psoriasis/drug therapy"[Mesh] OR "Psoriasis/economics"[Mesh] OR "Psoriasis/epidemiology"[Mesh] OR "Psoriasis/prevention and control"[Mesh] OR "Psoriasis/statistics and numerical data"[Mesh] OR "Psoriasis/therapy"[Mesh] OR "Arthritis, Psoriatic/drug therapy"[Mesh] OR "Arthritis, Psoriatic/epidemiology"[Mesh] OR "Arthritis, Psoriatic/prevention and control"[Mesh] OR "Arthritis, Psoriatic/therapy"[Mesh]) AND ("Cohort Studies"[Mesh] OR "Epidemiologic Studies"[Mesh] OR "Follow-Up Studies"[Mesh] OR "Longitudinal Studies"[Mesh] OR "Prospective Studies"[Mesh] OR "Registries"[Mesh] OR "Clinical Trials, Phase IV as Topic"[Mesh] OR "open label"[All Fields] OR "observational").

Titles and abstracts from the initial search were reviewed to identify relevant papers. A full paper review was then conducted on all those that met the general inclusion criteria. Reference lists of relevant studies were also hand searched to identify additional data. Contact with authors was not deemed necessary for the questions posed in this systematic review. Of those papers thought to be eligible, data on study characteristics were extracted and tabulated. Information collected included study design, objectives, patients, outcome measures, results, statistical methods and funding sources.

A quality assessment was conducted on all included studies. Although there are now guidelines on the reporting of observational studies (Strengthening the Reporting of Observational Epidemiological studies - STROBE)[25] which guide the author how to present their data, there are no consensus guidelines on quality assessment of such studies. A multitude of tools exist that claim to assess the validity of published observational studies[26]. We devised our own quality assessment tool based on a number of papers including the Downs and Black score system[27], the STROBE statement[25] and a recent systematic review of measures for assessing quality and susceptibility to bias in observational studies[26]. Each study was assessed against a list of 18 questions outlined in table 1. All results were summarised descriptively.

**Table 1:** Quality assessment tool

Item	Question
**Patients/selection bias**	1) Is the hypothesis/aim/objective of the study clearly described?
	2) Are the characteristics of the patients included in the study clearly described?
	3) Is the patient sample representative of patients treated in routine clinical practice?
	4) Is there information on possibility of selection bias present in study?

**Interventions**	5) Are the interventions of interest clearly described? *Treatments should be clearly described. In non-treatment related observational studies the characteristics under study should be clearly described.*

**Comparison**	6) Was a comparison group identified and clearly defined?

**Outcomes**	7) Are the main outcomes to be measured clearly described in the Introduction or Methods section? *If the main outcomes are first mentioned in the Results section, the question should be answered no.*
	8) Were the main outcome measures used accurate (valid and reliable)? *For studies where the outcome measures are clearly described, the question should be answered yes. For studies which refer to other work or that demonstrates the outcome measures are accurate, the question should be answered as yes.*
	9) Have all important adverse events that may be a consequence of the intervention been reported? *This should be answered yes if the study demonstrates that there was a comprehensive attempt to measure adverse events. (A list of possible adverse events is provided).*

**Reported findings/statistical analysis**	10) Are the main findings of the study clearly described? Simple outcome data (including *denominators and numerators) should be reported for all major findings so that the reader can check the major analyses and conclusions.(This question does not cover statistical tests which are considered below)*
	11) Does the study provide estimates of the random variability in the data for the main outcomes? *In non normally distributed data the inter-quartile range of results should be reported. In normally distributed data the standard error, standard deviation or confidence intervals should be reported. If the distribution of the data is not described, it must be assumed that the estimates used were appropriate and the question should be answered yes.*
	12) Were the statistical tests used to assess the main outcomes appropriate? *The statistical techniques used must be appropriate to the data. For example nonparametric methods should be used for small sample sizes. Where little statistical analysis has been undertaken but where there is no evidence of bias, the question should be answered yes. If the distribution of the data (normal or not) is not described it must be assumed that the estimates used were appropriate and the question should be answered yes.*

**Confounding**	13) Are the distributions of principal confounders in each group of subjects to be compared clearly described? A list of principal confounders is provided.
	14) Was there adequate adjustment for confounding in the analyses from which the main findings were drawn?

**Losses to follow-up**	15) Were losses of patients to follow-up reported?
	16) Were losses of patients to follow-up taken into account? *If the numbers of patients lost to follow-up are not reported, the question should be answered as unable to determine. If the proportion lost to follow-up was too small to affect the main findings, the question should be answered yes.*

**Power**	17) Was a sample size calculation reported?
	18) Did the study have sufficient power to detect a clinically important effect where the probability value for a difference being due to chance is less than 5%?*Sample sizes have been calculated to detect a difference of x% and y%.*

## Results

**Figure 1:**
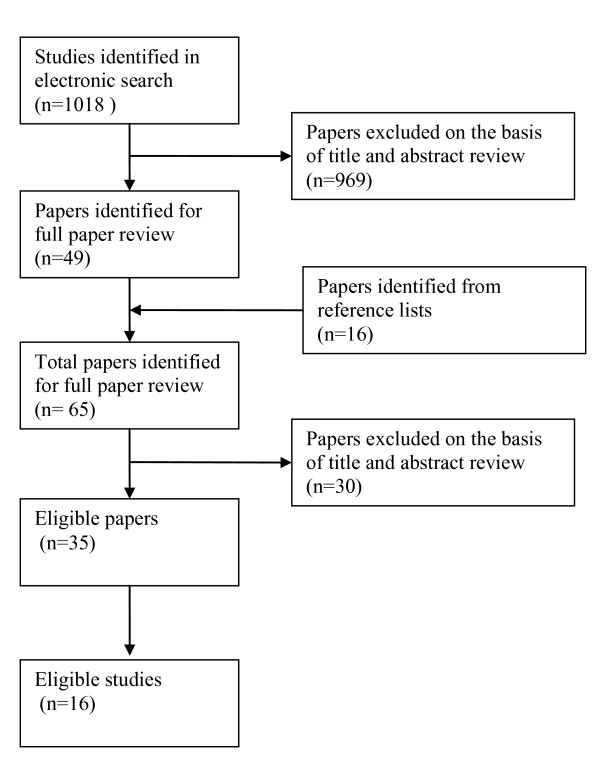
**Flow diagram of included studies**.

A total of 1018 papers were identified from the combination of searches (Figure 1). Fifty-eight papers were obtained for full paper review, of which 35 papers were identified as eligible for inclusion into the review. Reasons for exclusion included study design was experimental[28-43], sample size too small[44], the patients included were originally from a clinical trial[3,45-51], the study involved retrospective identification of patients[14,15,52-54] and the study was just a description of medications used with no attempt to assess outcomes[55].

The thirty-five papers relate to 16 observational studies, of which five were registry studies (Table 2). Nine were studies of psoriasis, six of psoriatic arthritis and one combined both conditions. Of the ten treatment-related observational studies only four evaluated biological agents; with the other six examining traditional therapies. Only two provided a comparison between two treatments. Of the six non-treatment related observational studies a number of characteristics of psoriasis and psoriatic arthritis were examined including mortality, morbidity, disease progression, cost of illness and aspects of HRQOL. Follow-up periods ranged from 3 months to 26 years.

**Table 2:** Characteristics of Included Studies

Primary reference, Study, Country (secondary references)	Objectives	Design	Sample size	Patient population	Follow-up
**Treatment-related observational studies**					

Driessen 2008[68], Radboud University Registry, The Netherlands (Driessen 2009[69])	Efficacy and tolerability of etenercept and efalizumab	Registry	118	Psoriasis	24 weeks

Fortune 2003[6], PUVA Study, UK and Ireland	Role of psychological distress on PUVA treatment outcomes	Cohort	112	Plaque psoriasis (chronic)	2 years

Lecha 2005[70], Spanish tacalcitol ointment study	Efficacy and tolerability of tacalcitol	Cohort	556	Psoriasis (moderate)	2 months

Naldi 2008[71], Psocare, Italy	Effect of BMI on clinical response to systemic treatment	Cohort	2368	Plaque psoriasis	3 years

Paul 2003[72] Cyclosporine study, Europe and Canada	Incidence of malignancies in cyclosporine treated patients	Cohort	1252	psoriasis - severe	5 years

Wahl 2005[73], Climate therapy study, Norway	Effectiveness of climate therapy	Cohort	286	Psoriasis	8 months

Heiberg 2008[74], Norwegian register of disease modifying anti-rheumatic drugs, (Heiberg 2007[75])	Comparative effectiveness of TNF inhibitors vs. methotrexate monotherapy	Registry	526	Psoriatic arthritis	1 year

Kristensen 2008[76], South Swedish Arthritis Treatment Group register, (Gulfe 2009[77], Geborek 2002[78], Kristensen 2006[79])	Efficacy, utility and tolerability of TNF-inhibitors (etanercept, infliximab, adalimumab)	Registry	261	Psoriatic arthritis	7 years

Sparado 1997[80], Cyclosporin Study, Italy	Probability of continuing to take cyclosporine vs. other DMARDS	Cohort	172	Psoriatic arthritis	10 years

Saad 2009[81], British Society for Rheumatology Biologics Register, (Harrison 2009[82], Silman 2003[83])	Effectiveness and tolerability of TNF-inhibitors (etanercept, infliximab, adalimumab)	Registry	566	RA, psoriasis, psoriatic arthritis	1 year

**Non-treatment related observational studies**					

Carrascosa 2006[84], EPIDERMA cost of illness, Spain	Direct and indirect cost; relationship between cost and severity	Cohort	797	Psoriasis	1 year

Colombo 2008[11], Cost of illness, Italy	Direct and indirect cost; HRQOL; relationship between cost, HRQOL and severity	Cohort	150	Moderate to severe plaque psoriasis	3 months

Schmid-ott 2005[85], Bad Bentheim Rehabilitation Hospital, Germany	Relationship between the degree of stigmatisation and gender, skin symptoms, PASI and SPASI	Cohort	166	Psoriasis	1 year

Ali 2007[86], Husted 2007[2], Gladman 2009[16], Rohekar 2008[87], Toronto PsA clinic, Canada, (Chandran 2007[88], Gladman 1995[89], Gladman 1998[90], Gladman 1999 [91], Gladman 2001[92], Husted 2005[93], Wong 1997[94])	(1) Mortality associated with PsA; (2) relationship between physical functioning, disease activity and joint damage; (3) CVD associated with PsA; (4) malignancies associated with PsA	Cohort	382 to 680	Psoriatic arthritis	26 years

Kane 2003[95], St. Vincent's University study, Ireland, (Kane 2003a[96])	Clinical presentation, outcome and prognosis of early PsA	Cohort	129	Early psoriatic arthritis	2 years

Lindqvist 2008[97], SwePsA registry, Sweden, (Svensson 2002[98])	Factors associated with disease progression; outcome of treated and non-treated groups, comparison of outcomes with RA patients	Registry	135	Early psoriatic arthritis	2 years

PUVA, Psoralen Ultra-Violet A. TNF, Tumor Necrosis Factor. DMARD, disease-modifying anti-rheumatic drug. RA, rheumatoid arthritis. HRQOL, health-related quality of life.

Table 3 outlines the clinical, patient-reported and cost measurements described in each of the studies. The main clinical outcome measure used in the psoriasis studies was the Psoriasis area and severity index (PASI), with some studies using the self-administered version of this measurement (SPASI). In the psoriatic arthritis studies the most common clinical measurements were those relating to tender and swollen joint counts with three of the studies using the disease activity score-28 (DAS) based on 28 tender and swollen joint counts. Eleven studies incorporated patient-reported outcomes into their analysis. The most common patient-reported outcome was the health assessment questionnaire (HAS) used in six studies, followed by the SF-36 used in five studies. One study used a questionnaire on experiences with skin complaints (QES) and one used the dermatology life quality index (DLQI). Only two studies assessed health utilities either using the EQ-5D or the SF-6D and again only two studies reported information on costs.

**Table 3:** Clinical, patient-reported and cost measurements reported in included studies

Study (first author*)	Measurements		
	**Clinical**	**Patient-reported**	**Cost**

**Treatment-related observational studies**			

Driessen 2008[68]	PASI	-	-

Fortune 2003[6]	Time taken to achieve clearance of psoriasis	Psychological distress, alcohol intake, HADS	-

Lecha 2005[70]	psoriasis severity and area, global efficacy and tolerability	Patients' satisfaction [tools not described].	-

Naldi 2008[71]	PASI, BMI	-	-

Paul 2003[72]	Malignancies	-	-

Wahl 2005[73]	SPASI	SF-36, one item on QOL and one assessment of self-acceptance	-

Heiberg 2008[74]	DAS-28	HAQ; SF-36; SF-6D (utility)	-

Kristensen 2008[76]	DAS-28, erythrocyte sedimentation rate and C-reactive protein	EQ-5D (utility) HAQ, VAS-pain, VAS-global, global evaluation	-

Sparado 1997[80]	Type of therapy; drug continuation; Number of painful and swollen joints; remission	-	-

Saad 2009[81]	Drug persistence; DAS-28	HAQ adapted for UK use and SF-36	-

**Non-treatment related observational studies**			

Carrascosa 2006[84]	PASI		Direct, indirect

Colombo 2008[11]	PASI	SF-36, DLQI	Direct, indirect

Schmid-ott 2005[85]	PASI, SPASI	QES	-

Husted 2007[2]	Mortality; PASI; Duration of morning stiffness, and total numbers of actively inflamed joints; incidence of CVD; malignancies	HAQ	-

Kane 2003[95]	PASI, Ritchie Articular Index, EULAR swollen joint count, joint stiffness on waking.	HAQ	-

Lindqvist 2008[97]	66/68 joint counts, PASI, physician's global assessment of joint disease activity, and subclassification; remission	VAS, HAQ, SF-36	-

*Primary paper for the study or the most recent analysis of the cohort.

PASI, psoriasis area and severity index. HADS, hospital anxiety and depression scale. DAS-28, disease activity score-28. HAQ, health assessment questionnaire. VAS, visual analogue scale. BMI, body mass index. SPASI, self-administered PASI. QOL, quality of life, DMARDs, disease-modifying anti-rheumatic drugs. DLQI, dermatology life quality index. QES, questionnaire on experiences with skin complaints.

**Figure 2:**
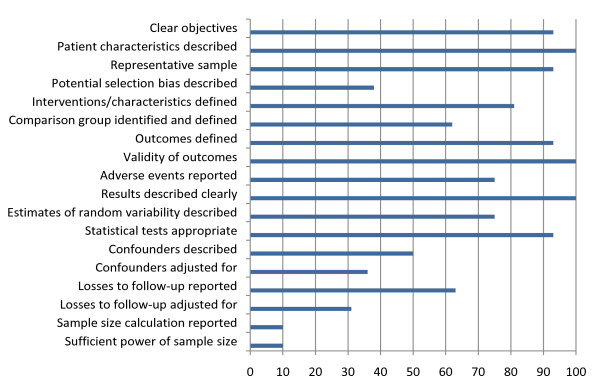
**Proportion (%) of studies meeting each of the quality assessment criteria**.

Overall the quality of the included cohort studies, measured against a checklist of 18 questions, ranged from 41% to 89% (taking into account the questions that were not applicable for certain studies) (Table 4). Figure 2 outlines the proportion of the 16 studies that met each of the quality assessment criteria. The studies in general did well on a number of quality assessment questions including having clear objectives, documenting selection criteria, providing a representative sample, defining interventions/characteristics under study, defining and using appropriate outcomes, describing results clearly and using appropriate statistical tests (where described). However, the studies fell short on a number of other quality assessment criteria. Only one study reported a sample size calculation or reported whether the sample size was sufficient for the study objectives. Only a third described potential selection bias. Around 50% described potential confounders and only a third adjusted for these potential confounders. Also, although over 60% reported losses to follow-up, less than a third made any adjustments for them in the analysis. Only around 60% of all studies identified and described a comparison group. Overall the proportion of studies meeting each quality assessment criteria ranged from 10% (sample size calculation and sufficient power) to 100% (patient characteristics described, validity of outcomes and results clearly described).

**Table 4:** Quality assessment of included studies

First author*	1	2	3	4	5	6	7	8	9	10	11	12	13	14	15	16	17	18	Sum
**Treatment-related observational studies**																			

Driessen 2008[66]	1	1	1	0	1	1	0	1	1	1	1	1	1	1	1	0	0	0	13

Fortune 2003[6]	1	1	1	0	1	1	1	1	0	1	1	1	1	1	1	1	1	1	16

Lecha 2005[68]	1	1	1	0	1	0	1	1	1	1	1	1	0	0	0	0	0	0	10

Naldi 2008[69]	1	1	0	1	0	1	1	1	na	1	1	1	0	0	0	0	0	0	9

Paul 2003[70]	1	1	1	0	1	1	1	1	0	1	1	1	1	0	0	0	0	0	11

Wahl 2005[71]	1	1	1	0	0	0	1	1	na	1	1	0	0	0	0	0	0	0	7

Heiberg 2008[72]	1	1	1	1	1	1	1	1	1	1	1	1	1	1	1	0	0	0	15

Kristensen 2008[74]	1	1	1	1	1	1	1	1	1	1	1	1	1	0	1	0	0	0	14

Sparado 1997[78]	1	1	1	0	0	1	1	1	1	1	0	1	0	0	1	1	0	0	11

Saad 2009[79]	1	1	1	1	1	0	1	1	1	1	1	1	1	1	0	0	0	0	13

**Non-treatment related observational studies**																			

Carrascosa 2006[82]	1	1	1	1	1	0	1	1	na	1	0	1	0	0	1	1	na	na	11

Colombo 2008[11]	1	1	1	0	1	1	1	1	na	1	0	1	0	0	1	1	na	na	11

Schmid-ott 2005[83]	0	1	1	1	1	0	1	1	na	1	0	1	0	0	1	1	na	na	10

Husted 2007[2]	1	1	1	0	1	1	1	1	na	1	1	1	1	1	1	0	na	na	13

Kane 2003[93]	1	1	1	0	1	0	1	1	na	1	1	1	0	0	1	0	na	na	10

Lindqvist 2008[95]	1	1	1	0	1	1	1	1	na	1	1	1	1	1	0	0	na	na	12

*Primary paper for the study or the most recent analysis of the cohort. NA, not applicable

## Discussion

Three important points can be concluded from this systematic review of large scale, prospective, observational studies conducted in patients with psoriasis or psoriatic arthritis. First, very few large-scale, prospective, observational studies have been conducted given the burden of these diseases on society and the recent introduction of biologic agents onto the market, with only two assessing a drug versus drug comparison. Psoriasis is the most prevalent autoimmune disease in the United States. It affects 125 million people worldwide (2-3% of the total population). Between 10 and 20% of people with psoriasis will develop psoriatic arthritis[56]. These conditions cause significant morbidity and have been associated with an increased risk of mortality compared to the general population[17]. They significantly affect a patient's HRQOL and ability to carry out normal activities[4] and the cost burden to society is substantial. In the United States psoriasis alone costs society $11.25 billion annually, with work loss accounting for 40% of this cost burden[57]. The recent introduction of biological therapies represent an important addition to the approaches used in the treatment of psoriasis and psoriatic arthritis, however very few studies have assessed these agents in real-life situations compared to the more traditional treatments where many patient and provider factors, not present in clinical trial environments, can impact on effectiveness[58]. Also, in some countries these agents are registered for use in specific target groups of patients where evidence of efficacy and safety are not provided by currently published clinical trials[58]. Finally, clinical trial data only provide short-term evidence of efficacy and safety in a highly selected group of patients. For all these reasons large scale, long-term observational studies in real-life situations are needed to guide appropriate clinical and policy decision making.

Second, given the importance of collecting health economic data in a real world environment[20], very few observational studies collected data on economic outcomes or patient utilities. In the general hierarchy of clinical evidence in healthcare decision making, RCT's remain the gold standard for evaluation. However, there are a number of situations where such studies may be unnecessary, inappropriate, impossible or inadequate[21]. The measurement of the effectiveness of a treatment, the longer-term outcomes of treatment (clinical and patient-reported), the true incidence of adverse events, and resource use associated with treatment and its side-effects are all situations where a RCT design is inadequate. RCT's often use patients, treatments and healthcare professionals that are all atypical and in addition are often short-term. Resource use and patient utilities observed in RCTs may not reflect that likely to be observed in regular clinical practice, not least because closer monitoring of patients in a trial may lead to events being detected and treated sooner than would otherwise be the case. This higher level of care may result in a small number of patients not experiencing high cost events that would be seen in everyday practice. In economic terms this is important since economic data is often highly skewed. The removal of a few observations with very high costs can have a large effect on overall health economic results. Also, RCTs are often conducted in specialist centres. The recorded resource consumption seen in the trial will therefore reflect the practice policies of this particular health care setting which may be very different to usual clinical practice. It is in such situations that observational cohort studies would provide more appropriate and informative health economic information if conducted and analysed rigorously.

Third, of those studies included in this review overall quality assessment was in general satisfactory, however the majority of studies failed to take into account and adjust for potential biases caused by lack of randomisation. Studies scored poorly on describing potential selection biases, identifying a comparison group, adjusting for confounders and losses to follow-up and providing adequate sample size calculations. The key question posed in cohort studies is the comparison of outcomes between two groups of patients (e.g. those responding to treatment vs. those not responding to treatment). Just over 60% of the studies in this review actually defined a comparison group, be it the general population or a more restricted internal or external population. For those studies not providing a comparison it is almost impossible to assess whether the results occurred by chance. Of those reporting a comparison group most studies reported potential selection bias however only half accounted for confounders and only a third accounted for losses to follow-up. In those studies not addressing these issues of potential bias, results are likely to have very low internal validity. Adjusting for the potential bias caused by lack of randomisation is critical to the validity of cohort studies[59-61].

When interpreting the results of this systematic review it is important to note three issues. First, it is difficult to systematically search for observational studies as search strategies that are both sensitive and specific do not exist for the major electronic databases. To overcome this problem we conducted a wide search and hand-searched reference lists of key papers. Second, consensus guidelines on the reporting of observational studies (STROBE) have only recently been introduced[25], therefore for many studies published prior to these guidelines it is often difficult to identify if the paper is a true observational study or not. Many studies stated they were observational, but in actual fact incorporated an experimental or 'open-label' element to them. Third, the cut-off of 100 patients to define large scale may have meant other important observational studies were excluded. However, only one study was excluded on the basis of sample size [44].

Large scale, prospective cohort studies are not the only non-randomised method for capturing real world health economic data. They are however, if conducted rigorously one of the best approaches to use, especially for non-rare outcomes over a relatively short period. A number of cross-sectional and case-control studies assessing cost, effectiveness and HRQOL have been conducted in patients with psoriasis and psoriatic arthritis. Cross-sectional studies are useful for assessing prevalence and describing specific characteristics of the disease, for example clinical and demographic characteristics, patient and provider perceptions of effectiveness, tolerability and compliance. However, unless they incorporate a retrospective element into their design, they are unable to distinguish between cause and effect and therefore are inappropriate for the measurement of effectiveness and health economic outcomes associated with an intervention. Retrospective elements to observational studies, for example retrospectively identifying patients or retrospective data collection (as in case control studies) introduces an additional level of bias and are therefore often used for more descriptive studies or hypothesis generation that can then be studied in a prospective observational study.

Looking outside of true observational designs to studies which are non-randomised but incorporate an experimental element to them, we find a number of 'open-label' trials, some aiming to assess longer-term outcomes and others aiming to assess effectiveness in a more naturalistic setting. These studies are not observational, although many claim to be. They are experimental in that patients have been selected for inclusion into the trial and administered the trial treatment. Given that these studies are not governed by any consensus guidelines on reporting or quality control, the potential for risk of error or bias is high and the results should be interpreted with caution. Included in these designs are 'open-label' extension studies. Patients represent a highly select group that have not only been selected on the basis of the original RCT, but are also those who have completed the randomized element to the trial and agreed to participate in the extension study. Such selection processes not only introduces significant bias, but also lowers even further the generalisability of the results to a wider population. In such studies the use of inferential statistics to allow for the possibility of sampling or random error to be the reason for the observed difference is crucial. However, in most extension studies assessing effectiveness in psoriasis or psoriatic arthritis no such inferential statistics have been carried out[62-64]. Also included are 'open-label' studies which adopt a non-randomised approach from the start of the study. Again these studies should be interpreted with caution for two main reasons; first, treatment is experimental and has therefore been selected by an investigator not independent from the study and second, the patient will know which treatment they are being given. Both actions will introduce inadvertent bias into the outcome assessment. Furthermore it is essential that such studies conform to the same rigorous methods expected of true observational studies in that the bias created from non-randomisation should be defined, explored and adjusted for. Currently, apart from one 'open-label' phase IV study assessing health economic outcomes which does account for confounding[34], most of the others don't[29,36,41].

## Conclusion

There is a clear need for well designed, large-scale, prospective observational studies in the field of psoriasis and psoriatic arthritis particularly to assess the impact of traditional and biological agents on economic and patient-reported outcomes and the factors that influence them, such as resistance and adherence, in a real world environment. Several population-based registries are currently being set up for both psoriasis and psoriatic arthritis[58,65-67]. However, while such registries will no doubt provide invaluable evidence on the long-term risks and benefits of new and old treatments, they fall short of providing adequate information on health economic outcomes. The recommended core datasets for registries include effectiveness measures[58,67], HRQOL measures[67], but no patient utilities and insufficient information with which to measure health care resources or work productivity. Future observational studies measuring such outcomes would be a welcome addition to the scientific literature in this area and would provide invaluable information to patients, clinicians and policy makers.

## Competing interests

This project was funded by Novartis Pharma AG. Dr. Langham and Dr. Ratcliffe are affiliated at PHMR Consulting. Mr. Goertz is affiliated at Novartis Pharma AG.

## Authors' contributions

HPG and MR were responsible for the conception and design of the study. SL and JL conducted the data analysis. SL, JL, HPG and MR interpreted the results. SL led drafting and revision of the manuscript. JL, HPG and MR contributed to revising the manuscript critically for important intellectual content. All authors approved the final version of the manuscript.

## Pre-publication history

The pre-publication history for this paper can be accessed here:

http://www.biomedcentral.com/1471-2288/11/32/prepub
